# Changes in Fetal Hemoglobin in Very Preterm Infants Born Small for Gestational Age: A Retrospective Observational Study

**DOI:** 10.3390/children13010117

**Published:** 2026-01-13

**Authors:** Carlo Dani, Federico Cipriani, Maria Ciavotta, Giulia Remaschi

**Affiliations:** 1 Department of Neurosciences, Psychology, Drug Research and Child Health, University of Florence, 50127 Florence, Italy; 2 Division of Neonatology, Careggi University Hospital of Florence, 50134 Florence, Italyremaschig@aou-careggi.toscana.it (G.R.)

**Keywords:** fetal hemoglobin, small for gestational age, intra uterine growth restriction, preterm infant

## Abstract

**Background:** Small-for-gestational-age (SGA) preterm infants are at higher risk for oxidative stress-related complications than appropriate-for-gestational-age (AGA) preterm infants. It has been proposed that HbF may be higher in SGA than in AGA infants due to fetal hypoxia. **Aim:** The aim of this study was to compare postnatal changes in HbF fractions in very preterm SGA and AGA infants and in subgroups of these patients who had been transfused with red blood cells (RBCs) or not. **Methods:** We studied 30 SGA and 60 AGA very preterm infants with a gestational age of 27.7 ± 1.6 and 27.9 ± 0.7 weeks, respectively. HbF fractions were recorded daily during the first week of life, at 14 ± 2, 21 ± 2, and 28 ± 2 days of life, and 36 weeks (±3 days of life) of postmenstrual age. **Results:** The HbF fractions measured from the first day of life to the 36th week of postmenstrual age decreased significantly in both the groups, without differences between the groups. Transfused and non-transfused SGA infants had similar values of HbF fraction, while transfused AGA infants had lower values of HbF fraction than non-transfused infants. **Conclusions:** HbF fraction decreased similarly in the postnatal period in very preterm SGA and AGA infants. RBC transfusions did not affect hemoglobin fraction (HbF) values in SGA infants but were associated with a reduction in HbF in AGA infants. These findings may be due to the effect of fetal preconditioning hypoxia in very preterm SGA infants.

## 1. Introduction

Fetal hemoglobin (HbF) has a greater affinity for oxygen and a greater capacity to deliver oxygen to tissues than adult hemoglobin (HbA) [1]. In term and preterm infants, HbF is 90% of total hemoglobin [1,2]. However, in preterm infants, it significantly decreases postnatally due to anemia of prematurity and frequent blood samplings [3]. As a result, very preterm infants often receive transfusions of adult donor blood, leading to replacement of HbF with HbA [4]. Therefore, the increased oxygen availability due to the reduced oxygen affinity of HbA may favor the development of harmful hyperoxia [5], the negative effects of which are amplified by the concomitant reduction in HbF which itself has an antioxidant effect [6,7] that is particularly beneficial in preterm infants who have an inefficient antioxidant enzyme system [8].

These findings support the reported negative association between HbF fraction and the risk of oxidative stress-related complications of prematurity, such as retinopathy of prematurity (ROP), bronchopulmonary dysplasia (BPD), and intraventricular hemorrhage (IVH) [2,8,9,10,11].

Small-for-gestational-age (SGA) preterm infants are at higher risk for complications than appropriate-for-gestational-age (AGA) preterm infants, including BPD [12,13], ROP [14,15], necrotizing enterocolitis (NEC) [15,16], and adverse neurodevelopmental outcome [17,18]. This may occur due to placental insufficiency and chronic hypoxia that impair organ development, as well as due to poor nutritional reserves and abnormal hormonal/metabolic pathways [19] that make SGA infants more fragile than AGA infants. However, the exact pathogenic mechanisms that worsen the outcomes of SGA infants are not fully understood. It is therefore plausible that HbF levels may influence the risk of complications of prematurity due to their antioxidant properties. However, the postnatal dynamics of HbF in SGA infants remains poorly studied. Although fetal hypoxia may lead to higher HbF levels in SGA infants compared to AGA infants, no studies have directly compared HbF levels between these groups.

Considering these premises, we hypothesized that the fractions of blood HbF determined during the first weeks of life may be higher in very preterm SGA infants than in very preterm AGA infants. To evaluate this hypothesis, we compared postnatal changes in HbF fractions in very preterm SGA and AGA infants as well as in subgroups stratified by red blood cell (RBC) transfusion status.

## 2. Materials and Methods

We conducted this retrospective observational study on preterm infants with gestational age < 30 weeks from January 2018 to June 2025 in the third-level neonatal intensive care unit of the Careggi University Hospital of Florence. SGA infants were identified as having birth weights below the 10th percentile for gestational age according to sex-specific standards [20], while AGA infants were defined as having a birth weight between the 10th and 90th percentile (inclusive) for gestational age. We chose this definition because it is one of the most widely used and its application in the clinical setting is simpler than a birth weight Z-score ≤ −2 standard deviations. Patients with major congenital malformations, chromosomal syndromes, inherited metabolic disorders, fetal hydrops, and lack of data were excluded, as were those with conditions known to affect HbF levels, such as hemoglobinopathies, hemolytic disease of the newborn, fetomaternal hemorrhage, previous intrauterine transfusion, and congenital infections. This study was conducted according to the Declaration of Helsinki and approved by the Tuscan Pediatric Ethics Committee (Approval code 133/2022, 5 May 2022). The committee deemed that informed consent was not required if it could not be obtained from all parents of the newborns included.

HbF levels and fractions were recorded daily during the first week of life, at 14 ± 2, 21 ± 2, and 28 ± 2 days of life, and 36 weeks (±3 days of life) of postmenstrual age. We also reported the lowest value (nadir value) of HbF measured within 36 weeks of postmenstrual age in both SGA and AGA preterm infants. Since HbF values and fractions are included in the blood gas analysis results, no additional blood sampling was required for this study. Moreover, HbF levels and fractions were not used for clinical decision.

For each newborn, we also recorded the following data from the electronic medical record: gestational age, birth weight, sex, antenatal steroids, type of delivery, clinical chorioamnionitis, Apgar score at 5 min, need and duration of noninvasive and invasive ventilation, patent ductus arteriosus requiring treatment based on echocardiographic findings [21], occurrence of sepsis, BPD, IVH, ROP, NEC > 2 grade, duration of hospitalization, and death.

Sepsis was diagnosed in the presence of a positive blood or cerebrospinal fluid culture and consistent concomitant clinical signs. BPD was defined as oxygen requirement at 36 weeks of postmenstrual age [22]. The severity of IVH was assessed using the classification of Papile et al. [23]. Bell’s criteria were used to diagnose NEC [24]. The ROP was evaluated in accordance with the International Classification of ROP [25].

A blood gas analyzer (ABL800, Radiometer Medical ApS, Copenhagen, Denmark) was used to measure HbF levels and fractions in arterial or capillary blood samples. HbF values were expressed as a percentage of total Hb and as blood concentration (g/dL). RBC transfusions were decided according to the guidelines of the Italian Society of Neonatology [26]. In each case adult, leukoreduced RBCs stored in SAG-M (adenine, 0.169 g/L; glucose, 9.0 g/L; mannitol, 5.25 g/L; sodium chloride, 8.77 g/L) without further concentration by centrifugation, less than 1 week old, and within 2 h of irradiation were used. A volume of 15 mL/kg of RBCs was transfused over 3–4 h. We assessed the number of patients requiring transfusions in the SGA and AGA infant groups, the volume and frequency of transfusions in the first week of life, between 8 and 28 days of life, and between 29 days of life and 36 weeks of postmenstrual age.

### 2.1. Primary and Secondary Endpoints

The primary endpoint of our study was the comparison of the HbF fraction values in SGA versus AGA preterm infants during the first week of life. Secondary endpoints were the comparison of HbF fractions in transfused and non-transfused SGA and AGA preterm infants at 1, 7, and 28 ± 2 days of life, and at 36 weeks (±3 days of life) of postmenstrual age.

### 2.2. Statistical Analysis

We previously reported that very preterm infants have an HbF fraction of 80 ± 11% in the first week of life [27]. Therefore, we calculated that a sample size of at least 30 infants in each group was needed to detect a statistically significant difference in HbF fraction of 10% between the SGA and AGA groups, with an 80% power at 0.05 level. Then, for each SGA case, two AGA infants born immediately before or after each SGA infant studied were selected, with a matching ratio of 2:1.

Clinical characteristics of infants were reported as mean and standard deviation, rate and percentage, or median and interquartile range (IQR). Statistical analysis was performed using Student’s *t*-test for parametric continuous variables, the Mann–Whitney U test for non-parametric continuous variables, and the Χ^2^ test for categorical variables. HbF fractions are reported as the mean and standard deviation and compared within groups by repeated-measures analysis of variance (ANOVA), and, after Mauchly’s test, indicated that the assumption of sphericity was not violated. A *p* < 0.05 was considered statistically significant.

To better evaluate the potential effect of RBC transfusions on HbF fractions, changes in HbF fraction were compared in transfused and non-transfused SGA and AGA infants. RBC transfusions were recorded for each patient and summarized as cumulative exposure within predefined study periods. Transfusion status was not treated as a time-dependent binary variable for the analysis of HbF, and HbF measurements obtained before the first transfusion were interpreted independently of subsequent transfusion exposure.

## 3. Results

We studied 30 SGA and 60 AGA preterm infants with a gestational age of 27.7 ± 1.6 and 27.9 ± 0.7 weeks, respectively. Their clinical characteristics are shown in Table 1. SGA infants had lower birth weight, required more mechanical ventilation, and had longer hospital stays than AGA infants.

**Figure 1:**
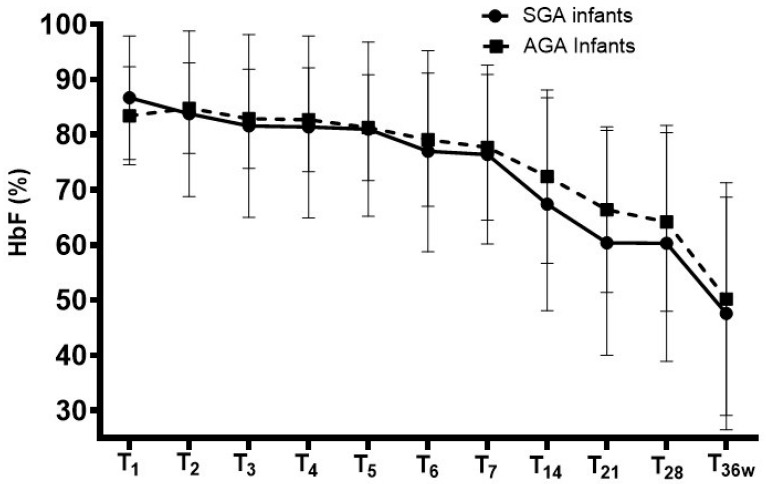
Changes in fetal hemoglobin (HbF) fractions in small-for-gestational-age (SGA) and appropriate-for-gestational-age (AGA) infants. The comparison of HbF fractions measured from the first day of life to the 36th week of postmenstrual age showed that it was similar between the groups and decreased significantly in both. Mean (±SD).

The HbF fractions measured from the first day of life to the 36th week of postmenstrual age did not show differences between SGA and AGA infants at selected timepoints. HbF fractions decreased significantly in both groups (Table 2, Figure 1).

Table 3 reports the comparison of Hb and HbF blood levels in SGA and AGA infant groups at the selected timepoints. Hb levels were lower on the 1st and 7th days of life in AGA than in SGA infants, while HbF levels were lower on the 7th day of life in AGA than in SGA infants.

**Figure 2:**
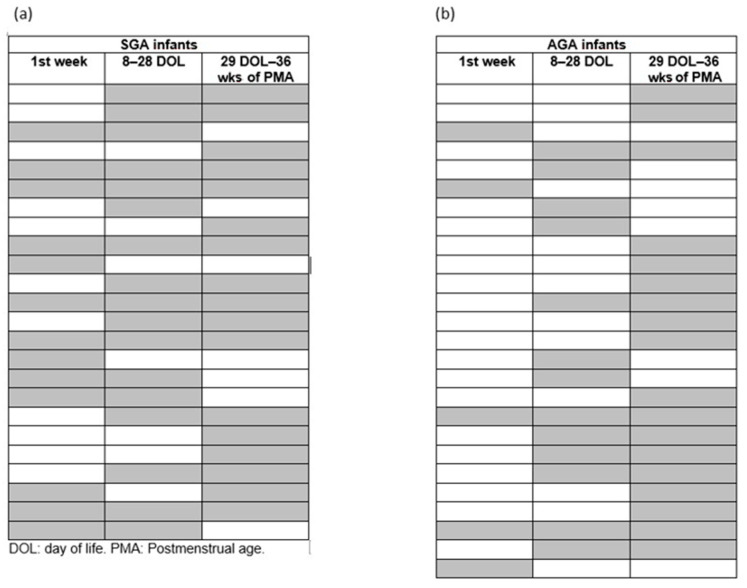
Distribution of red blood cell (RBC) transfusions in (**a**) small-for-gestational-age (SGA) and (**b**) appropriate-for-gestational-age (AGA) preterm infants during the 1st week of life, between 8 and 28 days of life (DOL), and between 29 DOL and 36 weeks of postmenstrual age (PCA). SGA infants required RBC transfusions more frequently than AGA infants, but the transfusion-to-patient ratio and the transfusion volumes were similar. Each row represents a patient, and the gray color indicates that he or she received at least one RBC transfusion during the period indicated in the column header.

SGA infants required RBC transfusions more frequently than AGA infants (80 vs. 43%, *p* < 0.001), but the transfusion-to-patient ratio and the transfusion volumes were similar (Table 2). The frequency of RBC transfusions was higher in SGA infants than in AGA infants during all three study periods considered, with a similar transfusion-to-patient ratio (Table 4, Figure 2).

**Figure 3:**
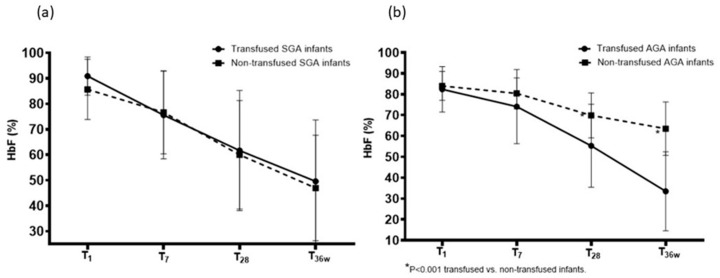
Comparison of fetal hemoglobin (HbF) fractions in (**a**) small-for-gestational-age (SGA) transfused and non-transfused infants and (**b**) appropriate-for-gestational-age (AGA) transfused and non-transfused infants. The HbF fractions were similar in transfused and non-transfused SGA infants but were lower in transfused than in non-transfused AGA infants. Mean (±SD).

Among SGA infants, 6 (20%) were not transfused and 24 (80%) were transfused. Both subgroups showed a gradual decrease in HbF fraction from day 1 of life to 36 weeks of postmenstrual age, and no differences were found at each time point (Table 5, Figure 3a). Among AGA infants, 26 (43%) were not transfused and 34 (57%) were transfused. These subgroups also showed a gradual decrease in HbF fraction from day 1 of life to 36 weeks of postmenstrual age, but the HbF fraction was lower at 28 days of life and at 34 weeks of postmenstrual age in transfused infants compared to non-transfused infants (Table 5, Figure 3b).

## 4. Discussion

This study is the first to compare blood HbF fraction values during the first weeks of life in very preterm SGA and AGA infants. We found that HbF fractions measured from the first day of life to 36 weeks of postmenstrual age were similar in SGA and AGA infants. Furthermore, we observed a similar progressive decline in HbF in both groups.

There are significant gaps in knowledge regarding postnatal changes in HbF levels in newborns. Roy et al. reported that the concentration of HbF fraction was lower in term than in preterm AGA infants and decreased at the rate of 2.4% per week [28]. Wilson et al. studied 159,215 infants and confirmed that HbF levels are inversely related to the gestational age and can be a useful tool for its postnatal prediction [29]. Bard et al. found in 13 preterm infants that HbF synthesis was unrelated to erythropoietin concentration and increased in patients with anemia of prematurity presumably due to chronic hypoxia [30]. The same mechanism was hypothesized by Bard et al. to explain the increased HbF synthesis in 12 preterm infants with BPD [31]. Moreover, Bard et al. demonstrated that at the postmenstrual age corresponding to term, there was no difference in the HbF fraction between 25 preterm infants (27–32 weeks of gestation) and 11 term infants, and they suggested that postnatal processes did not change the rate of transition from HbF to HbA [32].

There are no previous studies that specifically compared HbF fraction in SGA versus AGA very preterm infants, and it is difficult to compare our results with previous ones. Bard et al. measured the cord HbF in 39 infants ranging from 25 to 43 weeks of gestation and found that in SGA infants (whose gestational age ranged from 35 to 41 weeks), the HbF was higher than in AGA infants [33]. Moreover, Cochran-Black et al. studied 506 term and 30 late preterm infants and found that HbF fraction was similar in SGA and AGA late preterm infants but higher in term SGA infants than in term AGA infants [34]. However, these findings are not in agreement with Park et al. who reported that 25 SGA and 40 AGA term infants had similar HbF fractions at birth and at 1 month of life [35]. Therefore, our study fills a gap in the literature by studying the absolute HbF values in the postnatal period in very preterm AGA and SGA and demonstrating that they exhibit a similar postnatal reduction in HbF as previously described in late preterm infants [34].

It is known that the HbF-to-HbA switch occurs around the time of birth when erythropoiesis shifts from the fetal liver to the bone marrow [28,36]. A competitive interaction between repressors and activators at the promoters of genes (HBG1 and HBG2) on chromosome 11 that code for the gamma-globin chains regulates this switch [36,37]. It has been proposed that hypoxia can increase HbF synthesis and that activators of HBG1 and 2 include hypoxia-inducible factor (HIF) [36,38]. Consistently, it has been hypothesized that hypoxia may stimulate HbF synthesis in SGA preterm infants, inducing higher fractions of HbF than in AGA preterm infants [34,39]. Our results may seem to contradict this hypothesis, but in reality, it is likely that this mechanism cannot have detectable effects during fetal life, since HbF synthesis during this period is physiologically promoted in both SGA and AGA preterm infants, independently of hypoxia. Conversely, the effects of fetal hypoxia may become evident in SGA preterm infants in the postnatal period, influencing the mechanisms regulating HbF synthesis, as may occur in neonates requiring RBC transfusions.

We evaluated for the first time the possible association between RBC transfusions and HbF fraction in AGA and SGA infants since it is expected that their timing and volume may influence the nadir HbF level through a dilution effect. We found that transfused and non-transfused SGA infants had similar values of HbF fraction, while transfused AGA infants had lower values of HbF fraction than non-transfused infants at 28 days of life and 36 weeks of postmenstrual age, despite the similar patient-to-transfusion ratio and volume of transfusions. To explain these findings, we hypothesized that the chronic hypoxia experienced by SGA infants during intrauterine life may enhance their response to relative hypoxia due to anemia preceding RBC transfusions by increasing HbF synthesis and preventing its decrease. Indeed, hypoxia could stimulate the production of IGF1 which in turn would promote the synthesis of HbF as previously described [36,38]. This hypothesis could be supported by a study conducted on premature lambs, which highlighted how repeated episodes of hypoxia during fetal life determine an increase in the synthesis of HbF [40]. Furthermore, it is known that pre- and postnatal hypoxia has a protective preconditioning effect on the neonatal brain mediated precisely by the increased activity of HIF [41,42], which could also increase the synthesis of HbF. Therefore, it is plausible that this mechanism acts in SGA preterm infants as an effect of fetal preconditioning hypoxia, but not in AGA preterm infants, although further studies are needed to evaluate this hypothesis.

In our study, we found unexpectedly that Hb levels during the first week of life was lower in SGA than in AGA infants. This finding may be explained by several mechanisms related to impaired fetal growth. Placental insufficiency, frequently associated with SGA, can lead to reduced fetoplacental transfusion and a lower circulating blood volume at birth [43]. In addition, although chronic intrauterine hypoxia may stimulate erythropoiesis, this compensatory response may be insufficient or ineffective in the presence of prolonged placental dysfunction and limited nutrient availability [43]. Reduced maternal–fetal iron transfer and lower iron stores at birth may further contribute to lower hemoglobin concentrations in SGA infants [44]. Moreover, our SGA infants were born more frequently than AGA infants (90 vs. 67%) by cesarean section during which a fluid maternal overload might induce a decrease in Hb level [45].

The limitations of our study include its retrospective design and the small sample size, which limited the ability to perform subgroup analyses (e.g., according to different degrees or etiologies of growth restriction) and reduced the statistical power of the analyses in both transfused and non-transfused patients. Moreover, we could not associate the HbF fraction values to the risk of developing prematurity complications in SGA patients. However, our results could help stimulate further studies on this topic to confirm or refute our findings. The strengths of this study are its originality and the fact that HbF was measured serially from birth until 36 weeks of postmenstrual age. Indeed, in previous studies, HbF was measured only once at birth [28,31,33,34] or within 48 h of life [29], or twice, i.e., at birth and at term-equivalent age [32] or at one month of life [35].

In conclusion, we found that HbF fraction and levels similarly decreased in both SGA and AGA very preterm infants and that the reduction occurred similarly in the postnatal period. The reduction occurred similarly in the postnatal period. This result did not confirm the hypothesis that fetal hypoxia may stimulate HbF synthesis in SGA infants. Furthermore, we observed that RBC transfusions did not affect the HbF fraction in SGA infants, while they were associated with a decrease in HbF in AGA infants. We hypothesized that these findings might be the effect of fetal hypoxia-induced preconditioning, which might have influenced the regulation of postnatal HbF synthesis in SGA infants, but not in AGA infants. Further and larger studies are needed to confirm our findings and the possible role of HbF in preventing complications of prematurity in SGA and AGA preterm infants.

## Figures and Tables

**Table 1:** Clinical characteristics of small-for-gestational-age (SGA) and appropriate-for-gestational-age (AGA) infants. Mean (±SD) or percentage (%) or median and (IQR).

	SGA Infants (n = 30)	AGA Infants (n = 60)	*p*
Gestational age (wks)	27.7 ± 1.4	27.9 ± 0.7	0.367
Birth weight (g)	625 ± 143	1127 ± 192	<0.001
Female	15 (50)	28 (47)	0.765
Antenatal steroids	30 (100)	58 (97)	0.312
Cesarean section	27 (90)	40 (67)	0.017
Maternal clinical chorioamnionitis	2 (7)	2 (3)	0.470
Apgar score at 5 min	8 (7–8)	8 (8–9)	0.306
Noninvasive ventilation	27 (90)	59 (98)	0.071
Duration (days)	34 (8–50)	20 (6–28)	<0.001
Mechanical ventilation	16 (53)	13 (22)	0.002
Duration (days)	19 (6–31)	4 (3–20)	<0.001
Patent ductus arteriosus	12 (40)	23 (38)	0.521
Sepsis	11 (37)	16 (27)	0.329
Bronchopulmonary dysplasia	19 (63)	30 (50)	0.231
Intraventricular hemorrhage	7 (23)	20 (33)	0.329
Grade 1	3 (10)	5 (8)	
Grade 2	1 (3)	2 (3)	
Grade 3	2 (7)	13 (22)	
Grade 4	1 (3)	0	
Retinopathy of prematurity	6 (20)	5 (8)	0.170
Grade 1	0	4 (7)	
Grade 2	5 (17)	1 (2)	
Grade 3	1 (3)	0	
Necrotizing enterocolitis	2 (7)	3 (5)	0.745
Stage 1	2 (7)	1 (2)	
Stage 2	0	0	
Stage 3	0	2 (3)	
Duration of hospital stay (d)	89 ± 41	72 ± 22	0.012

**Table 2:** Comparisons of fetal hemoglobin (HbF) fractions in small-for-gestational-age (SGA) and appropriate-for-gestational-age (AGA) infants at selected timepoints and details on red blood cell (RBC) transfusions in the two groups. Mean (±SD) or percentage (%) or median and (IQR).

	SGA Infants (n = 30)	AGA Infants (n = 60)	*p*
1st day of life	86.7 ± 11.2	83.4 ± 8.9	0.133
2nd day of life	83.8 ± 15.0	84.8 ± 8.2	0.683
3rd day of life	81.6 ± 16.6	82.9 ± 9.0	0.631
4th day of life	81.4 ± 16.5	82.7 ± 9.4	0.635
5th day of life	81.0 ± 15.8	81.3 ± 9.6	0.911
6th day of life	77.0 ± 18.2	79.1 ± 12.1	0.516
7th day of life	76.4 ± 16.2	77.7 ± 13.2	0.685
*p* *	<0.001	<0.001	
14th day of life	67.4 ± 19.3	72.4 ± 15.7	0.191
21st day of life	60.4 ± 20.4	66.4 ± 15.0	0.194
28th day of life	60.3 ± 21.4	64.2 ± 16.2	0.316
36th weeks of postmenstrual age	47.6 ± 21.1	50.2 ± 21.1	0.685
*p* **	<0.001	<0.001	
Nadir level within 36 wks of postmenstrual age	35.0 ± 16.8	38.9 ± 17.4	0.314
Age at lowest level (days)	41 ± 11	41 ± 14	1.000
Patients transfused with RBCs Number of RBC transfusions Transfusion-to-patient ratio Volume of RBC (mL/kg) per infant	24 (80) 81 2 (1–3) 32.4 ± 46.7	26 (43) 55 1 (1–2) 32.9 ± 22.5	0.001 0.491 0.960

* *p* calculated with repeated-measures ANOVA test comparing values from day 1 to day 7 of life. ** *p* calculated with repeated-measures ANOVA test comparing values in the 31st, 34th, and 36th weeks of postmenstrual age.

**Table 3:** Comparisons of hemoglobin (Hb) and fetal hemoglobin (HbF) blood levels in small-for-gestational-age (SGA) and appropriate-for-gestational-age (AGA) infants at selected timepoints. Mean (±SD).

	**Hb (g/L)**					
	**Hb** **1st Day**	**Hb** **7th Day**	**Hb** **28th day**	**Hb** **31st wks**	**Hb** **36 wks**	***p***
SGA infants	15.5 ± 4.0	13.8 ± 2.2	11.3 ± 1.6	11.6 ± 2.9	10.1 ± 2.2	<0.001
AGA infants	17.2 ± 2.4	15.8 ± 2.4	11.7 ± 1.8	12.8 ± 2.5	9.8 ± 1.3	<0.001
*p*	0.014	<0.001	0.306	0.612	0.419	
	**HbF (g/L)**					
	**HbF** **1st day**	**HbF** **7th day**	**HbF** **28th day**	**HbF** **31st wks**	**HbF** **36 wks**	***p***
SGA infants	13.6 ± 4.1	10.7 ± 3.3	6.5 ± 2.9	7.2 ± 3.4	4.2 ± 2.6	<0.001
AGA infants	14.5 ± 2.9	12.5 ± 3.4	7.6 ± 2.7	8.3 ± 3.4	4.6 ± 2.1	<0.001
*p*	0.232	0.019	0.079	0.152	0.434	

**Table 4:** Number of patients transfused, number of transfusions, and transfusion-to-patient ratio in small-for-gestational-age (SGA) and appropriate-for-gestational-age (AGA) infants. Percentage (%) or median and IQR.

	1st Week			8–28 Days of Life			29 DOL-36 Wks of PCA		
	SGA	AGA	*p*	SGA	AGA	*p*	SGA	AGA	*p*
Patients	13 (43)	5 (8)	<0.001	17 (57)	13 (22)	<0.001	17(57)	18 (30)	0.014
Number of transfusions	23	6		30	25		28	24	
Transfusion-to-patient ratio	2 (1–3)	1 (1–1)	0.238	1 (1–2)	2 (1–3)	0.337	2 (1–2)	2 (1–2)	0.254

**Table 5:** Comparisons of fetal hemoglobin (HbF) fractions in transfused and non-transfused small-for-gestational-age (SGA) and appropriate-for-gestational-age (AGA) infants. Mean (±SD).

	SGA Infants				
	HbF 1st Day	HbF 7th Day	HbF 28th Day	HbF 36 Wks	*p*
Transfused (n = 24)	90.9 ± 7.5 ^a^	75.6 ± 17.2	61.7 ± 23.6	49.6 ± 24.1 ^b^	<0.001
Non-transfused (n = 6)	85.7 ± 11.8	76.7 ± 16.3	60.0 ± 21.3	47.0 ± 20.7 ^c^	<0.001
*p*	0.316	0.885	0.865	0.792	
	**AGA infants**				
Transfused (n = 26)	82.4 ± 10.9	74.1 ± 17.8	55.3 ± 19.9	33.5 ± 18.9	<0.001
Non-transfused (n = 34)	84.0 ± 6.9	80.4 ± 7.4	69.9 ± 10.8	63.5 ± 12.8	<0.001
*p*	0.490	0.067	<0.001	<0.001	

^a^ *p* = 0.003 vs. transfused AGA; ^b^ *p* = 0.001 vs. transfused AGA; ^c^ *p* = 0.012 vs. non-transfused AGA.

## Data Availability

All relevant data are within the manuscript.

## Abbreviations

The following abbreviations are used in this manuscript:

AGA	appropriate-for-gestational age
BPD	bronchopulmonary dysplasia
HbA	adult hemoglobin
HbF	fetal hemoglobin
IVH	intraventricular hemorrhage
NEC	necrotizing enterocolitis
RBC	red blood cell
ROP	retinopathy of prematurity
SGA	small-for-gestational age
